# Sustainability: Environmental Studies and Public Health

**DOI:** 10.3390/ijerph6102623

**Published:** 2009-10-09

**Authors:** Miklas Scholz

**Affiliations:** Institute for Infrastructure and Environment, School of Engineering, The University of Edinburgh, William Rankine Building, Mayfield Road, The King’s Buildings, Edinburgh EH9 3JL, Scotland, UK; E-Mail: m.scholz@ed.ac.uk; Tel.: +44-131-6506780; Fax: +44-131-6506554

This special issue ‘Sustainability: Environmental Studies and Public Health’ is part of the internationally leading ‘*International Journal of Environmental Research and Public Health’*. I was invited to be the guest editor, and to oversee the refereeing process and subsequent selection of timely, relevant and high quality papers highlighting particularly novel aspects concerned with sustainability issues in environmental studies.

Contributions that have a significant impact on solving public health problems were particularly encouraged. It follows that major public health issues, sustainable water management and environmental risk were topics of particular interest to the editorial team and reviewers. Ten papers comprising various scientific contributions including technical and critical review papers relevant for a world-wide audience were selected.

Three comprehensive critical review papers were published. Remoundou and Koundouri [1] reviewed the economic literature on the effects of environmental changes on public health, in both the developed and the developing world. They focused on the economic methodologies that are available for the evaluation of the effects of environmental changes on public health. Fleming *et al*. [2] explored the notion of ecological sustainability in the context of public health education and the contribution that Universities can make in creating environments that include ecologically sustainable practices. Finally, Rodriguez *et al*. [3] reviewed indirect potable reuse as a sustainable water supply alternative. This paper provided a state of the art review of water recycling for drinking purposes with emphasis on key membrane treatment processes. An overview of significant indirect potable reuse projects has been presented followed by a description of the epidemiological and toxicological studies evaluating any potential human health impacts.

Seven original papers were published in this special issue. Saarloos *et al*. [4] linked the built environment with key health issues. The paper proposes the use of an activity-based modeling approach for understanding and predicting, from the bottom up, how individuals interact with their environment and each other in space and time, and how their behaviors aggregate to population-level health outcomes. Staying with the built environment theme, Chen *et al*. [5] discussed the possibility of an autonomous house as a bio-hydrogen based energy self-sufficient approach. The proposed autonomous house combines energy-conserving, carbon emission-reducing passive design with active elements needed to maintain a comfortable environment.

The human health theme features also strongly in this special issue. For example, Markandya and Chiabai [6] valued climate change impacts on human health based on empirical evidence from the literature. This analysis served as a critical investigation of the methodologies used and identified research weaknesses and gaps. Furthermore, Ulhøi and Ulhøi [7] assessed the roles and responsibilities of hospitals and healthcare professionals. The paper concludes that arguments based on systems theory, environment, medicine, economics and innovation strongly urge hospitals to reconsider their present roles and environmental responsibilities.

Other papers had a clear international dimension. For example, Cross *et al*. [8] studied the export of vegetables from African countries to European markets, which often presents consumers with an ethical dilemma. If, for example, Ugandan produce enters UK markets, then consumers may wish to consider both the potential benefits that enhanced trade could offer Ugandan farmers compared with its potentially negative impact on UK workers. Furthermore, Priemus and Schutte-Postma [9] presented a discussion regarding the development of standards on particulate matter in the European Union, with specific reference to The Netherlands. Finally, Rigotto [10] assessed the role of non-governmental organizations in the process of industrial delocalization and socio-spatial redistribution of occupational and environmental risks.

The high diversity of excellent environmental and public health studies with a strong sustainability theme is likely to be of interest to a broad audience for decades to come. This special issue is not just a timely reference source for academics, but should particularly be of practical use for medical doctors, environmental scientists and engineers, and civil engineers concerned with public health engineering, environmental protection and sustainability.

It is a pleasure to thank the members of the Editorial Board, all authors and co-authors and all referees for their valuable contributions to this special issue. Moreover, this most interesting publication would not have been possible without the efficient support from the Journal Office.
